# Tris(2-amino-1,3-thia­zole-κ*N* ^3^)(7-oxa­bicyclo­[2.2.1]heptane-2,3-dicarboxyl­ato-κ^3^ *O* ^2^,*O* ^3^,*O* ^7^)cadmium(II) dihydrate

**DOI:** 10.1107/S1600536810027170

**Published:** 2010-07-17

**Authors:** Na Wang, Yi-Zhou Wu, Qiu-Yue Lin

**Affiliations:** a Zhejiang Key Laboratory for Reactive Chemistry on Solid Surfaces, Institute of Physical Chemistry, Zhejiang Normal University, Jinhua, Zhejiang 321004, People’s Republic of China; b College of Chemistry and Life Science, Zhejiang Normal University, Jinhua 321004, Zhejiang, People’s Republic of China; c College of Public Administration, Zhejiang University, Hangzhou, 310027, Zhejiang , People’s Republic of China

## Abstract

In the crystal structure of the title complex, [Cd(C_8_H_8_O_5_)(C_3_H_4_N_2_S)_3_]·2H_2_O, the Cd^II^ atom exhibits a slightly distorted octa­hedral CdO_3_N_3_ coordination, defined by the bridging O atom of the bicyclo­heptane unit, two O atoms from the carboxyl­ate groups and by three N atoms from three 2-amino­thia­zole ligands. Uncoordinated lattice water mol­ecules are also present in the crystal structure. N—H⋯O and O—H⋯O hydrogen-bonding inter­actions link the components into a three-dimensional structure.

## Related literature

For synthetic aspects, see: Yin *et al.* (2003 ▶). For background to 7-oxabicyclo­(2,2,1) heptane-2,3-dicarb­oxy­lic anhydride (nor­can­tharidin), see: Shimi *et al.* (1982 ▶).
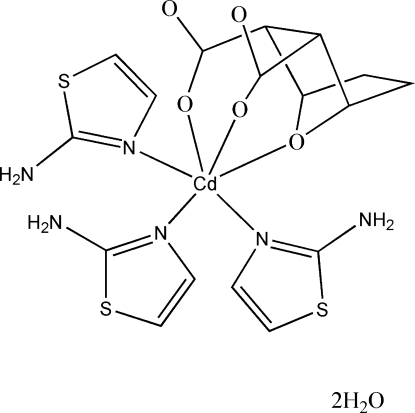


## Experimental

### 

#### Crystal data

[Cd(C_8_H_8_O_5_)(C_3_H_4_N_2_S)_3_]·2H_2_O
*M* *_r_* = 633.00
Monoclinic, 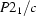

*a* = 9.6457 (3) Å
*b* = 9.9255 (3) Å
*c* = 25.4653 (9) Å
β = 101.980 (2)°
*V* = 2384.91 (13) Å^3^
*Z* = 4
Mo *K*α radiation
μ = 1.23 mm^−1^
*T* = 296 K
0.08 × 0.08 × 0.04 mm

#### Data collection

Bruker APEXII area-detector diffractometer
Absorption correction: multi-scan (*SADABS*; Sheldrick, 1996 ▶) *T*_min_ = 0.90, *T*_max_ = 0.95
19757 measured reflections
5480 independent reflections
2777 reflections with *I* > 2σ(*I*)
*R*_int_ = 0.100

#### Refinement

*R*[*F* ^2^ > 2σ(*F* ^2^)] = 0.057
*wR*(*F* ^2^) = 0.119
*S* = 1.01
5480 reflections
319 parameters
6 restraints
H atoms treated by a mixture of independent and constrained refinement
Δρ_max_ = 0.80 e Å^−3^
Δρ_min_ = −0.69 e Å^−3^

### 

Data collection: *APEX2* (Bruker, 2006 ▶); cell refinement: *SAINT* (Bruker, 2006 ▶); data reduction: *SAINT*; program(s) used to solve structure: *SHELXS97* (Sheldrick, 2008 ▶); program(s) used to refine structure: *SHELXL97* (Sheldrick, 2008 ▶); molecular graphics: *SHELXTL* (Sheldrick, 2008 ▶); software used to prepare material for publication: *SHELXL97*.

## Supplementary Material

Crystal structure: contains datablocks I, global. DOI: 10.1107/S1600536810027170/wm2366sup1.cif

Structure factors: contains datablocks I. DOI: 10.1107/S1600536810027170/wm2366Isup2.hkl

Additional supplementary materials:  crystallographic information; 3D view; checkCIF report

## Figures and Tables

**Table 1:** Selected bond lengths (Å)

Cd1—O4	2.268 (4)
Cd1—N4	2.302 (5)
Cd1—N2	2.305 (5)
Cd1—O2	2.312 (4)
Cd1—N6	2.341 (5)
Cd1—O1	2.467 (4)

**Table 2:** Hydrogen-bond geometry (Å, °)

*D*—H⋯*A*	*D*—H	H⋯*A*	*D*⋯*A*	*D*—H⋯*A*
N1—H1*A*⋯O2	0.86	2.04	2.867 (6)	161
N1—H1*B*⋯O3^i^	0.86	2.19	2.931 (6)	144
N3—H3*B*⋯O4	0.86	2.00	2.803 (7)	156
N3—H3*C*⋯O1*W*^ii^	0.86	2.14	2.965 (7)	160
N5—H5*B*⋯O1	0.86	2.15	2.917 (7)	149
N5—H5*C*⋯O3^iii^	0.86	2.46	3.177 (7)	141
N5—H5*C*⋯O2*W*^iv^	0.86	2.47	3.052 (9)	125
O1*W*—H1⋯O5^v^	0.86 (2)	1.97 (2)	2.817 (6)	174 (7)
O1*W*—H2⋯O3^vi^	0.85 (2)	2.03 (2)	2.865 (6)	168 (6)
O2*W*—H3⋯O5^vii^	0.86 (6)	2.25 (6)	2.996 (9)	145 (9)
O2*W*—H4⋯O5	0.88 (7)	2.12 (4)	2.962 (10)	162 (12)

Symmetry codes: (i) 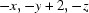; (ii) 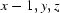; (iii) 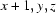; (iv) 
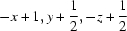
; (v) 
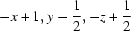
; (vi) 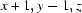; (vii) 
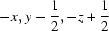
.

**Fig. 1.**
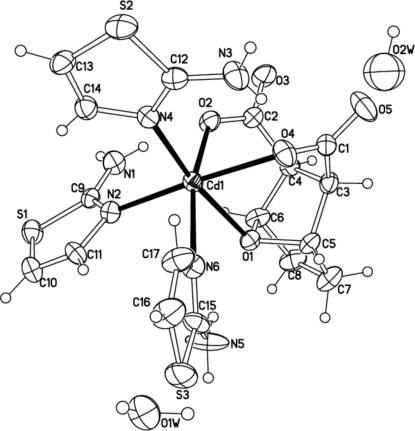
A view of the molecule of the title compound, showing the atom-labelling scheme and displacement ellipsoids drawn at the 30% probability level.

## supplementary crystallographic information

### Comment

7-oxabicyclo(2,2,1) heptane-2,3-dicarboxylic anhydride (norcantharidin), a traditional Chinese drug, has a great inhibitive effect on various cancer cells (Shimi *et al.*, 1982) which makes norcantharidin and its derivatives interesting compounds. Cadmium acetate can react with 2-aminothiazole and disodium demethylcantharate to form the title compound, [Cd(C_8_H_8_O_5_)(C_3_H_4_N_2_S)_3_]^.^2H_2_O.

The Cd^II^ atom exhibits a slightly distorted octahedral CdO_3_N_3_ coordination (Fig. 1), defined by the bridging O atom of the bicycloheptane unit, two O atoms from the carboxylate groups and by three N atoms from three different 2-aminothiazole ligands. O4, N6, N2 and O2 define the equatorial plane; O1 and N4 are in the axial positions. The bond angle O1—Cd1—N4 of 171.27 (14)° is indicative of the polyhedral distortion. Owing to the binding of the bridging oxygen atom to the Cd^II^ atom, two six-membered rings (Cd1—O1—C6—C4—C2—O2) and (Cd1—O1—C5—C3—C1—O4) are created. In addition, a seven-membered ring (Cd1—O2—C2—C4—C3—C1—O4) is formed which helps to stabilize the complex.

Uncoordinated lattice water molecules are also present in the crystal structure. N—H···O and O—H···O hydrogen-bonding interactions link the components into a three-dimensional structure.

### Experimental

Disodium demethylcantharate was prepared according to literature procedures (Yin *et al.*, 2003). Cadmium acetate, disodium demethylcantharate and 2-aminothiazole were dissolved in 15 ml distilled water. The mixture was sealed in a 25 ml Teflon-lined stainless vessel and heated at 443 K for 3 d, then cooled slowly to room temperature. Crystal suitable for X-ray diffraction were obtained.

### Refinement

The H atoms bonded to C and N atoms were positioned geometrically and refined using a riding model [aromatic C—H = 0.93 Å, aliphatic C—H = 0.97–0.98 Å and N—H = 0.86 Å, *U*_iso_(H) = 1.2*U*_eq_ of the carrier atom]. The H atoms of the water molecule were located in difference Fourier maps and were refined with O—H distance restraints of 0.85 (2) and *U*_iso_(H) = 1.5Ueq(O).

### Crystal data

**Table:**

[Cd(C_8_H_8_O_5_)(C_3_H_4_N_2_S)_3_]·2H_2_O	*F*(000) = 1280
*M**_r_* = 633.00	*D*_x_ = 1.763 Mg m^−^^3^
Monoclinic, *P*2_1_/*c*	Mo *K*α radiation, λ = 0.71073 Å
Hall symbol: -P 2ybc	Cell parameters from 1039 reflections
*a* = 9.6457 (3) Å	θ = 1.6–27.6°
*b* = 9.9255 (3) Å	µ = 1.23 mm^−^^1^
*c* = 25.4653 (9) Å	*T* = 296 K
β = 101.980 (2)°	Block, colorless
*V* = 2384.91 (13) Å^3^	0.08 × 0.08 × 0.04 mm
*Z* = 4	

### Data collection

**Table:**

Bruker APEXII area-detector diffractometer	5480 independent reflections
Radiation source: fine-focus sealed tube	2777 reflections with *I* > 2σ(*I*)
graphite	*R*_int_ = 0.100
φ– and ω–scans	θ_max_ = 27.6°, θ_min_ = 1.6°
Absorption correction: multi-scan (*SADABS*; Sheldrick, 1996)	*h* = −12→12
*T*_min_ = 0.90, *T*_max_ = 0.95	*k* = −12→11
19757 measured reflections	*l* = −32→33

### Refinement

**Table:**

Refinement on *F*^2^	Primary atom site location: structure-invariant direct methods
Least-squares matrix: full	Secondary atom site location: difference Fourier map
*R*[*F*^2^ > 2σ(*F*^2^)] = 0.057	Hydrogen site location: inferred from neighbouring sites
*wR*(*F*^2^) = 0.119	H atoms treated by a mixture of independent and constrained refinement
*S* = 1.01	*w* = 1/[σ^2^(*F*_o_^2^) + (0.0267*P*)^2^] where *P* = (*F*_o_^2^ + 2*F*_c_^2^)/3
5480 reflections	(Δ/σ)_max_ < 0.001
319 parameters	Δρ_max_ = 0.80 e Å^−^^3^
6 restraints	Δρ_min_ = −0.69 e Å^−^^3^

### Special details

**Table:**

Geometry. All e.s.d.'s (except the e.s.d. in the dihedral angle between two l.s. planes) are estimated using the full covariance matrix. The cell e.s.d.'s are taken into account individually in the estimation of e.s.d.'s in distances, angles and torsion angles; correlations between e.s.d.'s in cell parameters are only used when they are defined by crystal symmetry. An approximate (isotropic) treatment of cell e.s.d.'s is used for estimating e.s.d.'s involving l.s. planes.
Refinement. Refinement of *F*^2^ against ALL reflections. The weighted *R*-factor *wR* and goodness of fit *S* are based on *F*^2^, conventional *R*-factors *R* are based on *F*, with *F* set to zero for negative *F*^2^. The threshold expression of *F*^2^ > σ(*F*^2^) is used only for calculating *R*-factors(gt) *etc*. and is not relevant to the choice of reflections for refinement. *R*-factors based on *F*^2^ are statistically about twice as large as those based on *F*, and *R*- factors based on ALL data will be even larger.

### Fractional atomic coordinates and isotropic or equivalent isotropic displacement parameters (Å^2^)

**Table:**

	*x*	*y*	*z*	*U*_iso_*/*U*_eq_	
Cd1	0.30795 (4)	0.70542 (5)	0.098525 (16)	0.03617 (15)	
S1	0.40386 (19)	0.91245 (19)	−0.06279 (6)	0.0540 (5)	
S2	0.07421 (19)	0.28302 (19)	0.04108 (8)	0.0595 (5)	
S3	0.8009 (2)	0.5619 (2)	0.15111 (8)	0.0719 (6)	
O1	0.4020 (4)	0.9069 (4)	0.15068 (14)	0.0396 (10)	
O2	0.1101 (4)	0.8429 (4)	0.08458 (15)	0.0445 (11)	
O3	−0.0541 (4)	0.9542 (5)	0.11418 (16)	0.0617 (14)	
O4	0.2421 (5)	0.6604 (4)	0.17721 (17)	0.0540 (12)	
O5	0.1018 (5)	0.7150 (5)	0.23232 (19)	0.0780 (16)	
N1	0.1977 (5)	0.9502 (5)	−0.00787 (19)	0.0523 (15)	
H1A	0.1565	0.9321	0.0182	0.063*	
H1B	0.1637	1.0115	−0.0308	0.063*	
N2	0.3724 (5)	0.7869 (5)	0.02228 (18)	0.0393 (12)	
N3	0.1366 (5)	0.4106 (5)	0.1357 (2)	0.0573 (16)	
H3B	0.1682	0.4755	0.1573	0.069*	
H3C	0.0938	0.3437	0.1467	0.069*	
N4	0.2092 (5)	0.5071 (5)	0.06150 (18)	0.0398 (12)	
N5	0.6828 (6)	0.8058 (6)	0.1457 (3)	0.086 (2)	
H5B	0.6122	0.8606	0.1404	0.104*	
H5C	0.7680	0.8364	0.1540	0.104*	
N6	0.5362 (5)	0.6186 (5)	0.12915 (18)	0.0429 (13)	
C1	0.1932 (7)	0.7435 (7)	0.2067 (2)	0.0464 (18)	
C2	0.0712 (7)	0.9216 (6)	0.1179 (2)	0.0381 (15)	
C3	0.2499 (6)	0.8860 (7)	0.2105 (2)	0.0426 (16)	
H3A	0.2369	0.9256	0.2444	0.051*	
C4	0.1826 (6)	0.9839 (6)	0.1628 (2)	0.0401 (15)	
H4A	0.1420	1.0626	0.1774	0.048*	
C5	0.4063 (6)	0.8950 (7)	0.2080 (2)	0.0450 (17)	
H5A	0.4625	0.8183	0.2248	0.054*	
C6	0.3141 (6)	1.0263 (6)	0.1430 (2)	0.0442 (16)	
H6A	0.2937	1.0590	0.1059	0.053*	
C7	0.4675 (8)	1.0305 (8)	0.2285 (3)	0.069 (2)	
H7A	0.5701	1.0302	0.2344	0.083*	
H7B	0.4391	1.0553	0.2615	0.083*	
C8	0.4029 (7)	1.1250 (7)	0.1828 (3)	0.061 (2)	
H8A	0.4753	1.1685	0.1674	0.073*	
H8B	0.3440	1.1933	0.1945	0.073*	
C9	0.3141 (6)	0.8822 (6)	−0.0122 (2)	0.0368 (15)	
C10	0.5278 (7)	0.7915 (7)	−0.0356 (2)	0.0503 (17)	
H10A	0.6070	0.7679	−0.0492	0.060*	
C11	0.4922 (6)	0.7378 (6)	0.0074 (2)	0.0449 (17)	
H11A	0.5460	0.6696	0.0268	0.054*	
C12	0.1458 (6)	0.4122 (7)	0.0845 (3)	0.0451 (16)	
C13	0.1311 (7)	0.3632 (7)	−0.0100 (3)	0.0530 (18)	
H13A	0.1162	0.3322	−0.0452	0.064*	
C14	0.2003 (6)	0.4769 (7)	0.0078 (2)	0.0462 (17)	
H14A	0.2400	0.5323	−0.0146	0.055*	
C15	0.6606 (7)	0.6740 (7)	0.1417 (2)	0.0463 (18)	
C16	0.6826 (8)	0.4331 (8)	0.1364 (3)	0.074 (2)	
H16A	0.7062	0.3425	0.1352	0.088*	
C17	0.5514 (8)	0.4817 (8)	0.1269 (3)	0.064 (2)	
H17A	0.4731	0.4250	0.1190	0.076*	
O1W	0.9253 (5)	0.2156 (5)	0.15966 (19)	0.0631 (13)	
H1	0.922 (8)	0.211 (6)	0.1930 (10)	0.095*	
H2	0.926 (8)	0.134 (3)	0.150 (2)	0.095*	
O2W	0.1155 (7)	0.4230 (9)	0.2567 (3)	0.134 (3)	
H3	0.031 (5)	0.395 (10)	0.257 (5)	0.200*	
H4	0.101 (11)	0.503 (6)	0.242 (5)	0.200*	

### Atomic displacement parameters (Å^2^)

**Table:**

	*U*^11^	*U*^22^	*U*^33^	*U*^12^	*U*^13^	*U*^23^
Cd1	0.0370 (3)	0.0329 (3)	0.0398 (2)	0.0016 (2)	0.01077 (17)	−0.0009 (2)
S1	0.0660 (12)	0.0542 (13)	0.0446 (10)	−0.0029 (10)	0.0177 (9)	0.0082 (9)
S2	0.0546 (11)	0.0365 (12)	0.0867 (13)	−0.0082 (9)	0.0132 (10)	−0.0094 (10)
S3	0.0458 (12)	0.0877 (18)	0.0809 (14)	0.0263 (11)	0.0097 (10)	0.0158 (12)
O1	0.041 (3)	0.037 (3)	0.038 (2)	0.001 (2)	0.0041 (18)	0.004 (2)
O2	0.042 (3)	0.043 (3)	0.046 (2)	0.006 (2)	0.004 (2)	−0.011 (2)
O3	0.038 (3)	0.080 (4)	0.061 (3)	0.024 (3)	−0.004 (2)	−0.011 (3)
O4	0.068 (3)	0.041 (3)	0.059 (3)	−0.004 (2)	0.029 (2)	0.002 (2)
O5	0.088 (4)	0.076 (4)	0.088 (4)	−0.015 (3)	0.060 (3)	−0.012 (3)
N1	0.055 (4)	0.052 (4)	0.049 (3)	0.013 (3)	0.009 (3)	0.013 (3)
N2	0.046 (3)	0.032 (3)	0.040 (3)	0.004 (3)	0.009 (2)	0.000 (3)
N3	0.078 (4)	0.040 (4)	0.062 (4)	−0.014 (3)	0.033 (3)	0.000 (3)
N4	0.042 (3)	0.034 (3)	0.046 (3)	0.000 (2)	0.013 (2)	−0.001 (3)
N5	0.032 (3)	0.055 (5)	0.167 (7)	−0.012 (3)	0.007 (4)	0.022 (5)
N6	0.047 (4)	0.034 (4)	0.047 (3)	0.007 (3)	0.008 (2)	−0.002 (3)
C1	0.040 (4)	0.062 (6)	0.037 (4)	0.001 (3)	0.008 (3)	−0.001 (3)
C2	0.044 (4)	0.028 (4)	0.039 (4)	0.003 (3)	0.002 (3)	0.003 (3)
C3	0.047 (4)	0.045 (5)	0.035 (3)	0.000 (3)	0.007 (3)	−0.008 (3)
C4	0.036 (4)	0.038 (4)	0.046 (4)	0.007 (3)	0.009 (3)	−0.011 (3)
C5	0.043 (4)	0.051 (5)	0.035 (3)	0.000 (3)	−0.006 (3)	−0.003 (3)
C6	0.043 (4)	0.026 (4)	0.058 (4)	−0.001 (3)	−0.002 (3)	0.002 (3)
C7	0.068 (5)	0.074 (6)	0.056 (4)	−0.006 (4)	−0.011 (4)	−0.013 (4)
C8	0.057 (5)	0.041 (5)	0.077 (5)	−0.011 (4)	0.000 (4)	−0.013 (4)
C9	0.043 (4)	0.030 (4)	0.036 (3)	−0.003 (3)	0.006 (3)	−0.003 (3)
C10	0.058 (4)	0.050 (5)	0.048 (4)	0.007 (4)	0.024 (3)	0.004 (4)
C11	0.046 (4)	0.041 (5)	0.054 (4)	0.001 (3)	0.025 (3)	−0.002 (3)
C12	0.039 (4)	0.039 (5)	0.056 (4)	0.002 (3)	0.007 (3)	0.000 (3)
C13	0.052 (4)	0.049 (5)	0.056 (4)	−0.010 (4)	0.006 (3)	−0.013 (4)
C14	0.049 (4)	0.047 (5)	0.043 (4)	−0.001 (3)	0.012 (3)	0.001 (3)
C15	0.035 (4)	0.048 (5)	0.058 (4)	0.008 (3)	0.013 (3)	0.017 (4)
C16	0.066 (6)	0.058 (6)	0.087 (6)	0.021 (5)	−0.005 (4)	−0.016 (4)
C17	0.049 (5)	0.054 (6)	0.079 (5)	0.005 (4)	−0.006 (4)	−0.009 (4)
O1W	0.073 (3)	0.048 (3)	0.073 (3)	0.014 (3)	0.024 (3)	0.001 (3)
O2W	0.116 (6)	0.159 (8)	0.120 (5)	−0.020 (5)	0.011 (5)	0.032 (5)

### Geometric parameters (Å, °)

**Table:**

Cd1—O4	2.268 (4)	N6—C15	1.299 (7)
Cd1—N4	2.302 (5)	N6—C17	1.369 (8)
Cd1—N2	2.305 (5)	C1—C3	1.513 (9)
Cd1—O2	2.312 (4)	C2—C4	1.528 (8)
Cd1—N6	2.341 (5)	C3—C5	1.526 (8)
Cd1—O1	2.467 (4)	C3—C4	1.585 (8)
S1—C9	1.721 (6)	C3—H3A	0.9800
S1—C10	1.734 (6)	C4—C6	1.520 (8)
S2—C13	1.709 (7)	C4—H4A	0.9800
S2—C12	1.740 (6)	C5—C7	1.517 (9)
S3—C16	1.702 (8)	C5—H5A	0.9800
S3—C15	1.730 (6)	C6—C8	1.536 (8)
O1—C6	1.446 (6)	C6—H6A	0.9800
O1—C5	1.457 (6)	C7—C8	1.524 (9)
O2—C2	1.268 (6)	C7—H7A	0.9700
O3—C2	1.235 (6)	C7—H7B	0.9700
O4—C1	1.270 (7)	C8—H8A	0.9700
O5—C1	1.233 (7)	C8—H8B	0.9700
N1—C9	1.335 (7)	C10—C11	1.327 (8)
N1—H1A	0.8599	C10—H10A	0.9300
N1—H1B	0.8601	C11—H11A	0.9300
N2—C9	1.332 (7)	C13—C14	1.342 (8)
N2—C11	1.378 (7)	C13—H13A	0.9300
N3—C12	1.325 (7)	C14—H14A	0.9300
N3—H3B	0.8600	C16—C17	1.328 (9)
N3—H3C	0.8600	C16—H16A	0.9300
N4—C12	1.323 (7)	C17—H17A	0.9300
N4—C14	1.385 (7)	O1W—H1	0.855 (19)
N5—C15	1.325 (8)	O1W—H2	0.849 (19)
N5—H5B	0.8600	O2W—H3	0.86 (6)
N5—H5C	0.8601	O2W—H4	0.88 (7)
			
O4—Cd1—N4	91.47 (16)	C6—C4—H4A	109.4
O4—Cd1—N2	170.80 (16)	C2—C4—H4A	109.4
N4—Cd1—N2	96.67 (17)	C3—C4—H4A	109.4
O4—Cd1—O2	83.02 (15)	O1—C5—C7	101.5 (5)
N4—Cd1—O2	100.56 (15)	O1—C5—C3	102.9 (4)
N2—Cd1—O2	91.26 (16)	C7—C5—C3	110.8 (6)
O4—Cd1—N6	92.84 (16)	O1—C5—H5A	113.5
N4—Cd1—N6	95.89 (18)	C7—C5—H5A	113.5
N2—Cd1—N6	90.59 (17)	C3—C5—H5A	113.5
O2—Cd1—N6	163.12 (16)	O1—C6—C4	103.6 (5)
O4—Cd1—O1	79.80 (14)	O1—C6—C8	101.7 (5)
N4—Cd1—O1	171.27 (14)	C4—C6—C8	110.3 (5)
N2—Cd1—O1	92.05 (14)	O1—C6—H6A	113.4
O2—Cd1—O1	78.69 (13)	C4—C6—H6A	113.4
N6—Cd1—O1	84.48 (15)	C8—C6—H6A	113.4
C9—S1—C10	89.6 (3)	C5—C7—C8	102.5 (5)
C13—S2—C12	89.6 (3)	C5—C7—H7A	111.3
C16—S3—C15	89.0 (4)	C8—C7—H7A	111.3
C6—O1—C5	95.5 (4)	C5—C7—H7B	111.3
C6—O1—Cd1	116.9 (3)	C8—C7—H7B	111.3
C5—O1—Cd1	113.9 (3)	H7A—C7—H7B	109.2
C2—O2—Cd1	127.7 (4)	C7—C8—C6	101.2 (5)
C1—O4—Cd1	126.9 (4)	C7—C8—H8A	111.5
C9—N1—H1A	119.3	C6—C8—H8A	111.5
C9—N1—H1B	120.7	C7—C8—H8B	111.5
H1A—N1—H1B	120.0	C6—C8—H8B	111.5
C9—N2—C11	109.5 (5)	H8A—C8—H8B	109.4
C9—N2—Cd1	130.8 (4)	N2—C9—N1	123.5 (5)
C11—N2—Cd1	119.7 (4)	N2—C9—S1	114.1 (5)
C12—N3—H3B	122.2	N1—C9—S1	122.4 (5)
C12—N3—H3C	117.7	C11—C10—S1	109.4 (5)
H3B—N3—H3C	120.0	C11—C10—H10A	125.3
C12—N4—C14	110.1 (5)	S1—C10—H10A	125.3
C12—N4—Cd1	128.1 (4)	C10—C11—N2	117.4 (6)
C14—N4—Cd1	121.7 (4)	C10—C11—H11A	121.3
C15—N5—H5B	120.1	N2—C11—H11A	121.3
C15—N5—H5C	119.9	N4—C12—N3	125.1 (6)
H5B—N5—H5C	120.0	N4—C12—S2	113.7 (5)
C15—N6—C17	109.2 (6)	N3—C12—S2	121.2 (5)
C15—N6—Cd1	133.1 (4)	C14—C13—S2	110.6 (5)
C17—N6—Cd1	116.9 (4)	C14—C13—H13A	124.7
O5—C1—O4	123.9 (7)	S2—C13—H13A	124.7
O5—C1—C3	118.0 (6)	C13—C14—N4	116.1 (6)
O4—C1—C3	118.1 (6)	C13—C14—H14A	122.0
O3—C2—O2	122.1 (5)	N4—C14—H14A	122.0
O3—C2—C4	118.5 (5)	N6—C15—N5	124.2 (6)
O2—C2—C4	119.4 (5)	N6—C15—S3	114.7 (5)
C1—C3—C5	113.5 (5)	N5—C15—S3	121.0 (5)
C1—C3—C4	116.0 (5)	C17—C16—S3	109.7 (6)
C5—C3—C4	100.6 (5)	C17—C16—H16A	125.1
C1—C3—H3A	108.8	S3—C16—H16A	125.1
C5—C3—H3A	108.8	C16—C17—N6	117.3 (7)
C4—C3—H3A	108.8	C16—C17—H17A	121.3
C6—C4—C2	111.7 (5)	N6—C17—H17A	121.3
C6—C4—C3	100.7 (4)	H1—O1W—H2	104 (3)
C2—C4—C3	116.0 (5)	H3—O2W—H4	103 (10)
			
O4—Cd1—O1—C6	−96.4 (4)	C1—C3—C4—C6	−122.3 (5)
N2—Cd1—O1—C6	79.4 (4)	C5—C3—C4—C6	0.7 (6)
O2—Cd1—O1—C6	−11.5 (4)	C1—C3—C4—C2	−1.6 (8)
N6—Cd1—O1—C6	169.7 (4)	C5—C3—C4—C2	121.4 (5)
O4—Cd1—O1—C5	13.7 (3)	C6—O1—C5—C7	−57.5 (5)
N2—Cd1—O1—C5	−170.6 (3)	Cd1—O1—C5—C7	179.9 (4)
O2—Cd1—O1—C5	98.5 (3)	C6—O1—C5—C3	57.3 (5)
N6—Cd1—O1—C5	−80.2 (3)	Cd1—O1—C5—C3	−65.4 (5)
O4—Cd1—O2—C2	43.2 (5)	C1—C3—C5—O1	89.2 (6)
N4—Cd1—O2—C2	133.4 (5)	C4—C3—C5—O1	−35.5 (6)
N2—Cd1—O2—C2	−129.6 (5)	C1—C3—C5—C7	−163.0 (5)
N6—Cd1—O2—C2	−33.4 (8)	C4—C3—C5—C7	72.4 (6)
O1—Cd1—O2—C2	−37.7 (5)	C5—O1—C6—C4	−57.1 (5)
N4—Cd1—O4—C1	−139.6 (5)	Cd1—O1—C6—C4	63.2 (5)
O2—Cd1—O4—C1	−39.2 (5)	C5—O1—C6—C8	57.4 (5)
N6—Cd1—O4—C1	124.4 (5)	Cd1—O1—C6—C8	177.8 (3)
O1—Cd1—O4—C1	40.5 (5)	C2—C4—C6—O1	−89.0 (5)
N4—Cd1—N2—C9	103.8 (5)	C3—C4—C6—O1	34.7 (5)
O2—Cd1—N2—C9	3.0 (5)	C2—C4—C6—C8	162.8 (5)
N6—Cd1—N2—C9	−160.3 (5)	C3—C4—C6—C8	−73.5 (6)
O1—Cd1—N2—C9	−75.8 (5)	O1—C5—C7—C8	35.3 (6)
N4—Cd1—N2—C11	−78.4 (4)	C3—C5—C7—C8	−73.4 (6)
O2—Cd1—N2—C11	−179.2 (4)	C5—C7—C8—C6	−0.1 (7)
N6—Cd1—N2—C11	17.6 (4)	O1—C6—C8—C7	−35.5 (6)
O1—Cd1—N2—C11	102.1 (4)	C4—C6—C8—C7	74.0 (6)
O4—Cd1—N4—C12	−1.8 (5)	C11—N2—C9—N1	−179.0 (5)
N2—Cd1—N4—C12	−177.5 (5)	Cd1—N2—C9—N1	−0.9 (9)
O2—Cd1—N4—C12	−85.0 (5)	C11—N2—C9—S1	0.1 (6)
N6—Cd1—N4—C12	91.2 (5)	Cd1—N2—C9—S1	178.1 (3)
O4—Cd1—N4—C14	173.2 (4)	C10—S1—C9—N2	−0.6 (5)
N2—Cd1—N4—C14	−2.5 (4)	C10—S1—C9—N1	178.4 (5)
O2—Cd1—N4—C14	90.0 (4)	C9—S1—C10—C11	1.0 (5)
N6—Cd1—N4—C14	−93.8 (4)	S1—C10—C11—N2	−1.2 (7)
O4—Cd1—N6—C15	−109.7 (5)	C9—N2—C11—C10	0.7 (8)
N4—Cd1—N6—C15	158.5 (5)	Cd1—N2—C11—C10	−177.5 (4)
N2—Cd1—N6—C15	61.7 (6)	C14—N4—C12—N3	179.4 (6)
O2—Cd1—N6—C15	−34.5 (9)	Cd1—N4—C12—N3	−5.1 (9)
O1—Cd1—N6—C15	−30.3 (5)	C14—N4—C12—S2	0.2 (7)
O4—Cd1—N6—C17	81.7 (4)	Cd1—N4—C12—S2	175.7 (2)
N4—Cd1—N6—C17	−10.0 (5)	C13—S2—C12—N4	−0.8 (5)
N2—Cd1—N6—C17	−106.8 (4)	C13—S2—C12—N3	−180.0 (5)
O2—Cd1—N6—C17	156.9 (5)	C12—S2—C13—C14	1.1 (5)
O1—Cd1—N6—C17	161.2 (4)	S2—C13—C14—N4	−1.2 (7)
Cd1—O4—C1—O5	144.3 (5)	C12—N4—C14—C13	0.6 (8)
Cd1—O4—C1—C3	−35.5 (8)	Cd1—N4—C14—C13	−175.2 (4)
Cd1—O2—C2—O3	−154.3 (4)	C17—N6—C15—N5	178.0 (6)
Cd1—O2—C2—C4	28.5 (8)	Cd1—N6—C15—N5	8.8 (10)
O5—C1—C3—C5	145.8 (6)	C17—N6—C15—S3	0.1 (7)
O4—C1—C3—C5	−34.4 (7)	Cd1—N6—C15—S3	−169.1 (3)
O5—C1—C3—C4	−98.4 (7)	C16—S3—C15—N6	0.6 (5)
O4—C1—C3—C4	81.4 (7)	C16—S3—C15—N5	−177.3 (6)
O3—C2—C4—C6	−137.0 (6)	C15—S3—C16—C17	−1.2 (6)
O2—C2—C4—C6	40.3 (7)	S3—C16—C17—N6	1.5 (8)
O3—C2—C4—C3	108.4 (6)	C15—N6—C17—C16	−1.1 (9)
O2—C2—C4—C3	−74.4 (7)	Cd1—N6—C17—C16	170.1 (5)

### Hydrogen-bond geometry (Å, °)

**Table:**

*D*—H···*A*	*D*—H	H···*A*	*D*···*A*	*D*—H···*A*
N1—H1A···O2	0.86	2.04	2.867 (6)	161
N1—H1B···O3^i^	0.86	2.19	2.931 (6)	144
N3—H3B···O4	0.86	2.00	2.803 (7)	156
N3—H3C···O1W^ii^	0.86	2.14	2.965 (7)	160
N5—H5B···O1	0.86	2.15	2.917 (7)	149
N5—H5C···O3^iii^	0.86	2.46	3.177 (7)	141
N5—H5C···O2W^iv^	0.86	2.47	3.052 (9)	125
O1W—H1···O5^v^	0.86 (2)	1.97 (2)	2.817 (6)	174 (7)
O1W—H2···O3^vi^	0.85 (2)	2.03 (2)	2.865 (6)	168 (6)
O2W—H3···O5^vii^	0.86 (6)	2.25 (6)	2.996 (9)	145 (9)
O2W—H4···O5	0.88 (7)	2.12 (4)	2.962 (10)	162 (12)

Symmetry codes: (i) −*x*, −*y*+2, −*z*; (ii) *x*−1, *y*, *z*; (iii) *x*+1, *y*, *z*; (iv) −*x*+1, *y*+1/2, −*z*+1/2; (v) −*x*+1, *y*−1/2, −*z*+1/2; (vi) *x*+1, *y*−1, *z*; (vii) −*x*, *y*−1/2, −*z*+1/2.

## References

[bb1] Bruker (2006). *APEX2* and *SAINT* Bruker AXS Inc., Madison, Wisconsin, USA.

[bb2] Sheldrick, G. M. (1996). *SADABS* University of Göttingen, Germany.

[bb3] Sheldrick, G. M. (2008). *Acta Cryst.* A**64**, 112–122. 10.1107/S0108767307043930 18156677

[bb4] Shimi, I. R., Zaki, Z., Shoukry, S. & Medhat, A. M. (1982). *Eur. J. Cancer Clin. Oncol.* **18**, 785–789. 10.1016/0277-5379(82)90078-5 6891327

[bb5] Yin, F. L., Shen, J., Zou, J. J. & Li, R. C. (2003). *Acta Chim. Sin.* **61**, 556–561.

